# Exploring Interrater Disagreement on Essential Tremor Using a Standardized Tremor Elements Assessment

**DOI:** 10.1002/mdc3.13150

**Published:** 2021-02-12

**Authors:** Jos Becktepe, Felix Gövert, Bettina Balint, Christian Schlenstedt, Kailash Bhatia, Rodger Elble, Günther Deuschl

**Affiliations:** ^1^ Department of Neurology University Hospital Schleswig‐Holstein, Christian‐Albrechts‐University Kiel Germany; ^2^ Department of Clinical and Movement Neurosciences, Queen Square Institute of Neurology University College London London UK; ^3^ Department of Neurology University Hospital Heidelberg Heidelberg Germany; ^4^ Department of Neurology Southern Illinois University School of Medicine Springfield Illinois USA

**Keywords:** essential tremor, dystonic tremor, tremor, tremor classification

## Abstract

**Background:**

Patients with upper limb action tremor frequently exhibit additional neurological signs of uncertain significance. Clinicians vary in their interpretation, and interrater agreement on the final diagnosis is poor.

**Objectives:**

A new clinical tool for assessing the presence or absence of clinical signs that are important in axis‐1 classification of tremor patients is introduced: the Standardized Tremor Elements Assessment (STEA). Interrater agreement is determined, and signs leading to disagreement in the final diagnosis are identified.

**Methods:**

Three tremor‐focussed and one dystonia‐focussed movement disorder specialists rated 59 videos of patients with upper limb action tremor syndromes using STEA. Interrater agreements for final diagnosis and STEA items were calculated.

**Results:**

Interrater agreement regarding the final diagnosis was higher within the group of tremor specialists and poor between dystonia and tremor specialists. Greater agreement was found for items characterizing tremor than for signs of dystonia.

**Conclusions:**

Clinical signs leading to diagnostic disagreement were identified with STEA, and STEA should therefore be useful in future studies of diagnostic disagreement. The thresholds for considering neurological signs as soft versus significant for ataxia, parkinsonism, dystonia, etc. are critically important in tremor classification and must be studied across movement disorder subspecialties, not simply within a pool of tremor specialists.

Essential tremor (ET) is the most common isolated tremor disorder. However, the diagnosis of ET varies considerably among clinicians,^1^, ^2^ and heterogenous tremor disorders have been labeled as ET.^3^, ^4^

The new MDS tremor classification scheme^5^ adopted the same 2‐axis approach as in dystonia classifications^6^ (axis 1, clinical characteristics; axis 2, etiology) and introduced the concept of syndromes defined on the basis of axis‐1 features. ET is defined as an isolated tremor syndrome of bilateral upper limb action tremor of at least 3 years duration, with or without tremor in other locations (eg, head, voice, or lower limbs). In addition, a new classification ET plus was introduced for patients with the clinical characteristics of ET but with additional neurological signs of uncertain clinical significance (“soft signs”; eg, mildly unsteady tandem gait, questionably abnormal posturing of a body part, mild cognitive impairment, and questionable bradykinesia or rigidity).^5^ ET plus represents a borderline classification between ET and combined tremor syndromes.^7^

However, clinicians undoubtedly vary in their recognition and diagnostic use of questionable clinical signs. The thresholds for considering neurological signs as soft versus significant for ataxia, parkinsonism, dystonia, etc. are critically important in tremor classification. If a particular posture or movement is judged to be a sign of dystonia, the patient is classified as dystonic tremor or tremor associated with dystonia. However, if the posture or movement is judged to be questionably abnormal, the classification is ET plus. Recent studies have shown poor interrater reliability within the field of ET and ET plus syndromes as well as dystonia syndromes,^8^, ^9^, ^10^, ^11^, ^12^ and published case series suggest that clinicians vary in their interpretation of clinical signs that are relevant to tremor classification.^1^, ^2^ Several factors may contribute to this. Overall clinical experience, prior mentoring and a clinician's clinical/research focus may result in different interpretations of soft neurological signs, as well as hard signs of another movement disorder.^4^ This diagnostic uncertainty leads to the formation of less homogenous patient populations, and the treatment and research consequences of such uncertainty could be considerable. Nuanced phenotyping provides a basis for exploring genetic, pathological and pathophysiological features of ET and ET plus subtypes only if this phenotyping is consistent and accurate among clinicians. Consequently, the sources of disagreement in the clinical assessment of isolated and combined upper limb action tremor syndromes must be determined, and assessment tools for deeper clinical phenotyping of tremor patients are urgently needed.

The objectives of this study are (1) to introduce a new clinical assessment tool for documenting the presence or absence of neurological signs that are relevant to tremor classification; (2) to determine the interrater agreement for these neurological signs in a group of patients with ET, ET plus and combined tremor syndromes, with the main emphasis on dystonic tremor; and (3) to determine the extent to which the identification and interpretation of these neurological signs are influenced by a neurologist's clinical and research focus.

## Methods

Four movement disorder specialists from 3 movement disorder centres evaluated videotaped neurological examinations of 59 patients with the predominant clinical abnormality of tremor.^5^ Twenty seven neurological characteristics of each patient were assessed with a new standardized assessment called the Standardized Tremor Elements Assessment (STEA) (Table 1). These characteristics are relevant to the classification of patients being considered for the possible diagnosis of ET and are rated as absent (0), questionably present (0.5) or present (1) (see Supplement 1).

**TABLE 1:** Interrater agreement for the 27 items of the STEA

STEA items		All raters	Dystonia vs Tremor specialists	Tremor specialists
#	Assessment			
**1**	Head: rest tremor	strong	strong	strong
**2**	Head: postural tremor	strong	strong	strong
**3**	Head: intention tremor*			
**4**	Head: tremor regularity	moderate	moderate	strong
**5**	Head: mini jerks without tremor*			
**6**	Head: posturing	moderate	poor	strong
**7**	Head: geste maneuver	poor	poor	moderate
**8**	Hand: tremor asymetry	strong	moderate	strong
**9**	Hand: tremor regularity	moderate	moderate	strong
**10**	Isolated upper limb jerks*			
**11**	Abnormal posture of the trembling extremity	moderate	poor	strong
**12**	Abnormal posture of the non‐trembling extremity*			
**13**	Hand: task specific tremor*			
**14**	Extremity rest tremor	strong	moderate	strong
**15**	Rest tremor suppression by voluntary muscle activation*			
**16**	Hand: intention tremor	moderate	moderate	strong
**17**	Wing‐beating posture crescendo tremor	strong	strong	strong
**18**	Facial hyperkinesia	moderate	moderate	strong
**19**	Voice tremor	strong	strong	strong
**20**	Voice dystonia	strong	strong	strong
**21a**	Upper limb rapid alternating movement, right	strong	strong	strong
**21b**	Upper limb rapid alternating movement, left	strong	strong	strong
**22**	Upper limb dysmetria**			
**23**	Lower limb dysmetria	moderate	moderate	strong
**24**	Tandem gait	strong	strong	strong
**25**	Lower limb tremor	strong	moderate	strong
**26**	Trunk tremor	moderate	moderate	moderate
**27**	Bradykinesia	moderate	moderate	strong
Final diagnosis		moderate	poor	strong

* Too little variation of ratings to conduct Fleiss statistics.

** Too few patients with ratings>0 to conduct Fleiss statistics. K < 0.2 = poor (red), K 0.2‐0.6 = fair/moderate (yellow), K > 0.6 = strong (green).

The initial version of STEA was drafted by two of the authors (GD and RJE). The initial version of STEA was used in an assessment of 65 patient videos with non‐Parkinson tremor syndromes. These videos were not used in the present study and were evaluated by all but one (KB) of the authors. Discussions among the authors resulted in the present version of STEA and its standardized video protocol (Supplement 1 and 2).

Three of the 4 video raters are dedicated to tremor research (raters T1‐T3) while one rater is dedicated to dystonia research (rater D1). Patients were videotaped according to a standardized video protocol (see Supplement 2). Nineteen patients were videotaped at Southern Illinois University School of Medicine USA by one of the tremor specialists in this study and 40 patients were videotaped at Christian‐Albrechts‐University in Kiel Germany by the other two tremor specialists. The patients were all new referrals for action tremor syndromes. Patients with a Parkinson syndrome were excluded.

All patients gave written informed consent, and the study was approved by the Springfield Committee for Research Involving Human Subjects and the research ethics committee of the Christian‐Albrechts‐University Kiel.

### Statistics

Fleiss' quadratically weighted Kappa (K) was calculated to investigate the interrater reliability among all raters. To analyze the agreement between two raters, Cohen's Kappa was calculated. We considered kappa values ≤0 as no agreement, 0–0.2 as slight, 0.21–0.4 as fair, 0.41–0.6 as moderate, 0.61–0.8 as substantial, and 0.81–1 as almost perfect.^13^ Friedman test was performed to detect differences in STEA ratings among the 4 raters, and the significance level was corrected for multiple comparisons (*P* < 0.01).

## Results

### Interrater Agreement on Tremor Diagnosis

The overall interrater agreement among the 4 raters for tremor diagnosis (ET, ET plus or combined tremor syndrome) was only fair (Fleiss kappa 0.34, 95% CI 0.09 to 0.57). Table 2 summarizes the diagnoses of the 4 raters. The dystonia specialist diagnosed dystonic tremor syndromes in 79.7% of patients, and the tremor specialists diagnosed ET in more than 50%. Only 2 patients were classified as ET by all 4 raters, and no patient was rated as ET plus by all. Only 8 patients were diagnosed as combined tremor syndrome by all raters.

**TABLE 2:** Final diagnoses by each rater

	Rater			
Diagnosis	D1	T1	T2	T3
ET	2	36	30	34
ET plus	10	15	20	15
Combined tremor syndrome	47	8	9	10

D1 differed from T1, T2, and T3 in the distributions of patient diagnoses, but there was no statistical difference among T1, T2 and T3 (Friedman test, *P* < 0.00001). The Fleiss quadratically weighted Kappa (K) for all four raters was 0.34 (95% CI = 0.09 to 0.57), and the quadratically weighted Kappa (K) for the three tremor specialists was 0.83 (95% CI = 0.75 to 0.89).

Within the group of tremor specialists, the agreement in diagnosis was moderate to substantial: Cohen's K = 0.559, 0.602, and 0.768 for T1 vs T2, T1 vs T3, and T2 vs T3, with a mean kappa value of 0.643. By contrast, agreement between tremor and dystonia specialists was poor: Cohen's K = 0.027, −0.003, and 0.009 for D1 vs T1, D1 vs T2, and D1 vs T3, with a mean K of 0.011.

### Interrater Agreement on Items of STEA

Table 1 summarizes the interrater agreement for each item of STEA for all 4 raters, between dystonia and tremor specialists, and for the 3 tremor specialists. Items with substantial or almost perfect interrater agreement (K > 0.6) among all 4 raters were rest (STEA item 1) and postural (item 2) head tremor, asymmetry of hand tremor (item 8), extremity rest tremor (item 14), crescendo tremor in the wing‐beating posture (item 17), voice tremor (item 19), voice dystonia (item 20), rapid alternating movements of the upper limb (item 21), tandem gait ataxia (item 24), and lower limb tremor (item 25). Poor (K < 0.2) agreement among all raters was found for head tremor suppression with geste maneuver (item 7). Head posturing (item 6) and abnormal posture of a trembling extremity (item 11) had poor agreement between dystonia and tremor specialists but not among the tremor specialists. Thus, items relevant to dystonic tremor were the primary sources of interrater disagreement, leading to systematic classification (diagnosis) differences between dystonia and tremor specialists.

## Discussion

We have shown that the diagnosis of ET differs considerably among movement disorder specialists, although our raters made their diagnosis based on the MDS classification scheme.^5^ The interrater reliability is low for many neurologic signs that are suggestive or indicative of ET plus questionable signs of dystonia or a dystonia tremor syndrome. Key questions are why tremor classification differs so markedly among investigators and what is necessary to overcome this problem. These questions can only be answered with additional studies that include larger numbers of dystonia and tremor specialists.

We developed STEA in order to facilitate a standardized clinical assessment and documentation of neurological signs that are important in axis‐1 classification of tremor patients and to identify neurological signs that would lead to a diagnosis of ET plus or a combined tremor syndrome, rather than ET. STEA is not a severity scale, rather STEA is a tool for capturing aspects of the neurological exam that are important in differential diagnosis and that are not captured by tremor severity scales (eg, Fahn‐Tolosa‐Marin scale,^14^ the Essential Tremor Rating Assessment scale^15^). Additional studies are needed to determine if the current items of STEA are sufficient and if modification of items is needed. This will require broad collaboration among dedicated groups of movement disorder specialists, including those with special interest in ataxia, myoclonus and Parkinson syndromes. Collaboration among relevant study groups of the MDS is a logical path forward.

Although all raters strictly adhered to the new MDS tremor classification scheme,^5^ the overall agreement on the clinical diagnosis was only fair. Most items in STEA require examiners to judge whether a sign is absent (score = 0), questionable (score = 0.5) or present (score = 1). This is in keeping with the new tremor classification ET plus,^5^ which encourages clinicians to document signs of uncertain clinical significance. However, the ultimate utility of this approach is still uncertain.^9^, ^16^, ^17^, ^18^ It is clear that soft signs are common in patients previously classified as ET,^12^, ^19^ and patients with ET and ET plus or a combined dystonic tremor syndrome (eg, dystonic tremor) can have the same or different etiology.^20^, ^21^, ^22^ ET plus was introduced by the MDS Tremor Task Force in hopes that a deeper, careful phenotyping will facilitate the discovery of specific etiologies, and STEA was produced to facilitate this process.

We found a complex pattern of interrater agreement with a tendency towards better agreement for the tremor distribution and activation items and worse agreement for items related to dystonia. Poor agreement for items relevant to dystonia occurred because the tremor specialists were more likely to rate posturing of the trembling upper extremity (item 11) and abnormal posturing of the head (item 6) as soft signs, while the dystonia specialist was more likely to score the same posturing as definitely abnormal (Table S1). In some instances, dystonic posturing was rated by the dystonia specialist but not by the tremor experts. These results suggest that tremor and dystonia diagnoses are strongly influenced by a clinician's training, research interests, or clinical practice. Additional studies with larger numbers of specialists are needed to confirm this finding and to determine the extent to which disagreement can be resolved.

We hypothesize that interrater agreement can be increased by training and the development of consensus guidelines based on teaching video libraries. The poor agreement between dystonia and tremor specialists and the strong agreement among tremor specialists support this hypothesis. Our results suggest that dystonia specialists have lower thresholds for signs suggestive of dystonia. Interrater reliability among dystonia specialists should be determined in future studies. The diagnostic challenge of ET vs DT is well documented,^10^, ^12^ and the uncertain threshold and validity of subtle dystonic signs are equally well known.^8^ Full resolution of these issues will require diagnostic gold standards that do not exist except for dominantly‐inherited dystonias such as DYT‐TOR1A and DYT‐ANO3.^21^, ^23^

A limitation of our study is that all ratings were performed on standardized video exams, and the dystonia specialist did not examine any of the patients in person. Greater interrater agreement might have occurred if each rater had examined all patients personally, with freedom to employ personalized exam techniques. However, good agreement occurred among the tremor specialists even though they had not personally examined many of the patients. Research in movement disorders strongly relies on video examinations, and tools like STEA are needed for a more standardized and reliable video assessment of patients with tremor. STEA does not and cannot encompass all aspects of the neurological exam that might be important for diagnosis. In particular, cognitive symptoms and rigidity are not covered. STEA is a tool that should be used in conjunction with a complete history and neurological examination and should not be viewed as a comprehensive examination.

Another limitation is that only one dystonia specialist was among our group of raters, and the assessment of interrater reliability among dystonia specialists is an important topic for future studies. Disagreement on dystonic signs in tremor patients has been reported by other investigators and needs to be investigated fully.^12^

STEA was conceived as a tool for documenting the presence of neurologic signs that are relevant to the classification of tremor. STEA is not a rating scale per se, and total scores are not meaningful. However, it is likely that the number of items scored 0.5 is more important than the presence of a single 0.5 item. For example, suspicious “dystonic” hand posturing (eg, spooning and index finger pointing) may be too common in controls and other neurologic conditions to be considered incompatible with ET,^24^ but a 0.5 on this item and one or more other items may be diagnostically significant. By contrast, a single score of 1 on many STEA items is incompatible with the classifications ET and ET plus. Additional studies are needed to define the limits of consensus that can be achieved for each item of STEA and to determine if additional items are needed or if some items are not useful. Future studies will require the examination of large numbers of patients and healthy controls. The validity of soft or subtle signs of dystonia and the limits of normal for rapid alternating hand movements and tandem gait also require additional study.^25^

## Author Roles

1. Research project: A. Conception, B. Organization, C. Execution. 2. Statistical Analysis: A. Design, B. Execution, C. Review and Critique. 3. Manuscript Preparation: A. Writing of the first draft, B. Review and Critique.

JB: 1A, 1B, 1C, 2A, 2B, 3A, 3B.

FG: 1A, 1B, 1C, 2C, 3A, 3B.

BB: 1C, 3B.

CS: 2A, 2B.

KB: 1A, 1B, 3B.

RE: 1A, 1B, 1C, 2C, 3B.

GD: 1A, 1B, 1C, 3B.

## Disclosures

**Ethical Compliance Statement:** All patients gave written informed consent to participate in this study. The study was approved by the Springfield Committee for Research Involving Human Subjects (SCRIHS) and the research ethics committee of the Christian‐Albrechts‐University Kiel. We confirm that we have read the Journal's position on issues involved in ethical publication and affirm that this work is consistent with those guidelines.

**Funding Sources and Conflicts of Interest:** No specific funding was received for this work. The authors declare that there are no conflicts of interest relevant to this work.

**Financial Disclosures for the previous 12 months:** JB, FG, and CS have nothing to disclose. BB is supported by the Robert Bosch Foundation. KB has received consulting fees/ honoraria for speaking at meetings from Ipsen, Cavion, Retrophin, Merz, Allergan, Teva, and Ultragenyx pharma. He has grant support from Horizon 2020 EU grant. He receives royalty for books published by OUP and Cambridge Publishers. He has received stipend for editorial work for MDCP journal and has received honoraria from MDS to teach at International MDS meetings and summer/winter schools. RE has received consulting fees from Applied Therapeutics, Cavion, Cadent, Cydan, Jazz, Osmotica, Praxis Medicines and Sage. He receives research funds from the Kiwanis Neuroscience Research Foundation, Illinois‐Eastern Iowa District, and he is on the Medical Advisory Board of the International Essential Tremor Foundation and the Kiwanis Neuroscience Research Foundation, Illinois‐Eastern Iowa District. He has no other financial disclosures. GD received fees for lecturing from Boston Scientific and consulting fees from Boston Scientific, Cavion, Functional modulation. He receives funding for his research to his institution from the German Research Council, SFB 1261, B5 and Medtronic.

## Supporting information


**Table S1**. Distributions of STEA item scores for the four examining physicians and 59 patients. The Friedman test was performed for each STEA item. This is a nonparametric test of repeated measures (4 raters of 59 patients). Items with numbers in bold red font had a statistically significant (*P* < 0.01) Friedman test. A *P*‐value of 0.01 was used because of the multiple comparisons.

**Supplementary 1.** Standardized Tremor Elements Assessment (STEA) rating instructions.

**Supplementary 2.** Videotape exam protocol for STEA.
