# Supplementary material for: Exploring Interrater Disagreement on Essential Tremor Using a Standardized Tremor Elements Assessment
Source: Mov Disord Clin Pract. 2021 Feb 12;8(3):371–6. doi: 10.1002/mdc3.13150 (PMC8015892; doi:10.1002/mdc3.13150)
Supplement: Supplementary file 1 — Table S1. Distributions of STEA item scores for the four examining physicians and 59 patients. The Friedman test was performed for each STEA item. This is a nonparametric test of repeated measures (4 raters of 59 patients). Items with numbers in bold red font had a statistically significant (P < 0.01) Friedman test. A P‐value of 0.01 was used because of the multiple comparisons. [file MDC3-8-371-s001.docx]

**Examining interrater disagreement on essential tremor using a standardized tremor elements assessment**

Jos Becktepe MD^1^*, Felix Gövert MD^1^*, Bettina Balint MD^2,3^, Christian Schlenstedt PhD^1^, Kailash Bhatia FRCP, MD^2^, Rodger Elble MD, PhD^4^, Günther Deuschl MD, PhD^1^

^1^ Department of Neurology, University Hospital Schleswig-Holstein, Christian-Albrechts-University, Kiel, Germany

^2^ Department of Clinical and Movement Neurosciences, Queen Square Institute of Neurology, University College London, United Kingdom

^3^ Department of Neurology, University Hospital Heidelberg, Germany

^4^ Department of Neurology, Southern Illinois University School of Medicine, Springfield, USA

**Supplementary Table:** Distributions of STEA item scores for the four examining physicians and 59 patients. The Friedman test was performed for each STEA item. This is a nonparametric test of repeated measures (4 raters of 59 patients). Items with numbers in bold red font had a statistically significant (p<0.01) Friedman test. A p-value of 0.01 was used because of the multiple comparisons.

| STEA items | | Friedman test  p value | Number of patients rated 0 | | | | Number of patients rated 0.5 | | | | Number of patients rated 1 | | | |
| --- | --- | --- | --- | --- | --- | --- | --- | --- | --- | --- | --- | --- | --- | --- |
| # | Assessment |  | T1 | T2 | T3 | D1 | T1 | T2 | T3 | D1 | T1 | T2 | T3 | D1 |
| 1 | Head: rest tremor | 0.047 | 50 | 50 | 50 | 53 | 1 | 1 | 2 | 1 | 8 | 8 | 7 | 5 |
| 2 | Head: postural tremor | 0.06 | 22 | 26 | 24 | 19 | 6 | 6 | 7 | 14 | 31 | 27 | 28 | 26 |
| 3 | Head: intention tremor | 0.39 | 58 | 58 | 58 | 59 | 1 | 0 | 1 | 0 | 0 | 1 | 0 | 0 |
| 4 | Head: tremor regularity | **<0.00001** | 50 | 49 | 48 | **31** | 4 | 7 | 8 | **21** | 5 | 3 | 3 | **7** |
| 5 | Head: mini jerks without tremor | 0.71 | 58 | 58 | 58 | 59 | 1 | 1 | 1 | 0 | 0 | 0 | 0 | 0 |
| 6 | Head: posturing | **<0.00001** | 41 | 40 | 41 | **9** | 15 | 14 | 13 | **16** | 3 | 5 | 5 | **34** |
| 7 | Head: geste maneuver | **0.007** | 58 | 58 | 58 | 52 | 1 | 1 | 1 | **5** | 0 | 0 | 0 | **2** |
| 8 | Hand: tremor asymmetry | **0.00002** | 19 | 25 | 23 | **9** | 21 | 13 | 17 | **25** | 19 | 21 | 19 | **25** |
| 9 | Hand: tremor regularity | **<0.00001** | 50 | 49 | 48 | **31** | 4 | 7 | 8 | **21** | 5 | 3 | 3 | **7** |
| 10 | Isolated upper limb jerks | 0.92 | 58 | 57 | 57 | 58 | 1 | 1 | 2 | 0 | 0 | 1 | 0 | 1 |
| 11 | Abnormal posture of the trembling extremity | **<0.00001** | 46 | 37 | 41 | **12** | 11 | 19 | 17 | **20** | 2 | 3 | 1 | **27** |
| 12 | Abnormal posture of the non-trembling extremity | 0.28 | 56 | 58 | 57 | 58 | 2 | 1 | 1 | 1 | 1 | 0 | 1 | 0 |
| 13 | Hand: task-specific tremor | 0.03 | 59 | 59 | 59 | 56 | 0 | 0 | 0 | 3 | 0 | 0 | 0 | 0 |
| 14 | Extremity rest tremor | **<0.00001** | 40 | 38 | 38 | **25** | 10 | 10 | 11 | **8** | 9 | 11 | 10 | **26** |
| 15 | Rest tremor suppression by voluntary muscle activation | **0.00021** | 58 | 59 | 59 | **51** | 1 | 0 | 0 | **5** | 0 | 0 | 0 | **3** |
| 16 | Hand: intention tremor | **0.00014** | 37 | 29 | 32 | 21 | 20 | 28 | 26 | **35** | 2 | 2 | 1 | **3** |
| 17 | Wing-beating posture crescendo tremor | 0.73 | 46 | 45 | 46 | 44 | 4 | 5 | 5 | 6 | 9 | 9 | 8 | 9 |
| 18 | Facial hyperkinesia | **<0.00001** | 51 | 51 | 51 | **36** | 8 | 8 | 8 | **9** | 0 | 0 | 0 | **14** |
| 19 | Voice tremor | **0.00581** | **39** | 46 | 43 | 49 | **16** | 10 | 13 | 5 | **4** | 3 | 3 | 5 |
| 20 | Voice dystonia | 0.15 | 53 | 50 | 50 | 48 | 4 | 5 | 6 | 7 | 2 | 4 | 3 | 4 |
| 21 | Upper limb rapid alternating movement | 0.80 | 45 | 45 | 45 | 43 | 5 | 8 | 6 | 7 | 9 | 6 | 8 | 9 |
| 22 | Upper limb dysmetria | -- | -- | -- | -- | -- | -- | -- | -- | -- | -- | -- | -- | -- |
| 23 | Lower limb dysmetria | **<0.00001** | 58 | 57 | 58 | **49** | 0 | 1 | 0 | **8** | 1 | 1 | 1 | **2** |
| 24 | Tandem gait | **0.00852** | 37 | 37 | 35 | **30** | 7 | 8 | 7 | **10** | 15 | 14 | 17 | **19** |
| 25 | Lower limb tremor | 0.34 | 40 | 45 | 45 | 43 | 15 | 11 | 10 | 14 | 4 | 3 | 4 | 2 |
| 26 | Trunk tremor | **0.00556** | **53** | 56 | 56 | 59 | **5** | 3 | 2 | 0 | **1** | 0 | 1 | 0 |
| 27 | Bradykinesia | **0.00395** | 52 | 53 | 53 | **47** | 6 | 6 | 6 | **9** | 1 | 0 | 0 | **3** |

-- All ratings were 0 or were missing due to inadequate video examination
