# Supplementary material for: Exploring Interrater Disagreement on Essential Tremor Using a Standardized Tremor Elements Assessment
Source: Mov Disord Clin Pract. 2021 Feb 12;8(3):371–6. doi: 10.1002/mdc3.13150 (PMC8015892; doi:10.1002/mdc3.13150)
Supplement: Supplementary file 2 — Supplementary 1. Standardized Tremor Elements Assessment (STEA) rating instructions. [file MDC3-8-371-s003.docx]

**Standardized Tremor Elements Assessment (STEA)**

The purpose of this assessment is to document neurological signs that are viewed as being incompatible with the diagnosis of essential tremor. Particularly, the distinction between essential and dystonic tremor is addressed. This assessment is intended to serve as a supplement to severity scales that quantify tremor (e.g., UTRS; Fahn-Tolosa-Marin scale, TETRAS) but do not capture aspects of the neurological exam that may be important in differential diagnosis.

1) Rest tremor of the head - Present when the patient is Iying down and the head is resting with full support.

0 = absent

0.5 = uncertain

1 = present

2) Postural tremor of the head - Head tremor is postural if it occurs while the head is held against gravity without any particular task being performed.

0 = absent

0.5 = uncertain

1 = present

3) Intention tremor of the head - Present if the patient has increasing tremor amplitudes or jerkiness during actions like drinking, pointing with the finger to the nose or other activities that involve a targeted positioning task of the head.

0 = absent

0.5 = uncertain

1 = present

4) Head tremor: Regularity of head tremor^[[1]](#footnote-1)^

0 = Rhythmic

0.5 = Questionable arrhythmic or irregular jerky amplitude variations

1 = Definite jerkiness or irregular amplitude variations in at least one head position

5) Isolated mini-jerks of the head when tremor is not present ^[[2]](#footnote-2)^

0 = absent

0.5 = uncertain

1 = present

6) Head posturing (rotation in the sagittal, coronal or axial plane, close eyes and count backwards from 10) is rated as:

0 = Normal head posture

0.5 = Occasional mild asymmetric positioning of the head in any direction that is not interpreted as dystonia

1 = Dystonic movement or posturing in any direction

7) Head tremor suppression with a geste maneuver: Ask patient if he/she has discovered a way of suppressing tremor by touching some area of the head or neck. Also, explore with the patient the possibility that the head tremor is suppressed by a sensory trick. The patient should slightly touch the chin or cheek first with his left and then with his right hand. The hand should not forcefully press against the head (counterpressure). In some instances, geste maneuvers against other parts of the head are possible. If the examiner or patient believes a geste is present, it should be demonstrated in the video 3 times.

0 = No effect

0.5 = Uncertain effect

1 = Consistent reduction

8) Asymmetry of hand tremor - Asymmetry is rated as follows:

0 = Bilateral tremor with no appreciable asymmetry

0.5 = uncertain or inconsistent asymmetry that is always less than 1 point on the UTRS II scale one of the hand items

1 = Bilateral tremor with asymmetry of ≥1 point on the UTRS II scale one of the hand items

9) Regularity of hand tremor^1^

0 = Rhythmic

0.5 = Questionable arrhythmic or irregular jerky amplitude variations

1 = Definite jerkiness or irregular amplitude variations for less than half the time period

10) Isolated mini-jerks in an upper limb when tremor is not present^2^

0 = absent

0.5 = uncertain

1 = present

11) Abnormal or suspicious posturing^[[3]](#footnote-3)^ of a trembling extremity (arm, hand, fingers, leg, foot).

0 = No abnormal or asymmetric posturing

0.5 = Slight or inconsistent posturing that is not interpreted as dystonia (e.g., posturing to limit tremor amplitude)

1 = Definite dystonic posturing

12) Abnormal or suspicious posturing of a non-trembling extremity (e.g., arm, hands, fingers, leg, foot)

0 = No abnormal or asymmetric posturing

0.5 = Slight or inconstant asymmetric posturing that is not interpreted as definitely dystonic (e.g., casual asymmetry or (sub-) conscious positioning to limit tremor amplitude)

1 = Definite dystonic posturing

13) Task-specific tremor of the hands without dystonic posturing (if abnormal posturing is present this is covered under item 11)

0 = no task-specific tremor

0.5 = Tremor is barely visible (< 5 mm) in other locations and actions.

1 = Tremor is only seen during a specific task (i.e., writing or other task specific tremor occurs in isolation)

14) Extremity rest tremor

0 = no rest tremor of the upper or lower limbs

0.5 = questionable rest tremor (versus incomplete relaxation)

1 = rest tremor (when a limb is adequately supported and relaxed)

15) Suppression of rest tremor by voluntary muscle activation

0 = no suppression of rest tremor

0.5 = questionable suppression of rest tremor

1 = rest tremor is blocked during or before the movement

16) Upper extremity intention tremor

0= During finger-to-nose test (FTN) tremor amplitude is fairly similar throughout the movement (peak amplitude at target <50% greater than other points in the movement) or tremor is suppressed

0.5 = On FTN tremor present throughout, but tremor at target >50% greater than other points

1 = On FTN, tremor isolated to target

17) Crescendo postural tremor in the wing-beating position

0 = tremor amplitude in wing-beating does not increase appreciably over 20 seconds

0.5 = tremor amplitude in wing-beating increases less than 100% over 20 seconds

1 = tremor amplitude more than doubles over 20 seconds

18) Facial hyperkinesia (voice is rated separately)

0 = none

0.5 = eye blinking, grimacing of the lower face or other non-tremor facial contractions that are not definitely dystonic

1 = dystonic periocular movements, grimacing or jaw movements

19) Voice tremor

0 = none

0.5 = tremor only during “aaaah” or “eeee”

1 = tremor while speaking

20) Dystonia of voice

0 = none

0.5 = uncertain: hoarseness or choked voice or occasional arrhythmic voice breaks could be normal

1 = definite dystonic hoarseness, choked voice or voice breaks

21) Ataxia in patients with ET

Assessment of diadochokinesis. The paradigm is the standard test with repetitive clapping of one hand (palm alternating with dorsum) on the other palm. Forearm must be kept in a horizontal position, and palm should be held waist high. Disruption is defined as an interruption of the harmonic rhythm. One clapping cycle is defined as one palm/dorsum sequence. Clapping speed should be around 1/sec.

Count the number of obvious interruptions per 5 clapping cycles, three trials with each limb:

Right: _____ _____ _____ Left: _____ _____ _____

22) Upper extremity dysmetria during finger chasing task

0 = no dysmetria

0.5 = questionable dysmetria that may be difficult to discern from tremor

1 = definite dysmetria

23) Lower extremity dysmetria during heel-knee-shin test

0 = no dysmetria

0.5 = questionable dysmetria that may be difficult to discern from tremor

1 = definite dysmetria

24) Tandem gait tested in a distance of 10 consecutive tandem steps (heel to toe). The number of missteps^[[4]](#footnote-4)^ is counted for each of 3 trials:

Trial 1: _____ Trial 2: _____ Trial 3: _____

25) Lower limb tremor. Ask the patient while seated to extend each leg horizontally with ankle dorsiflexed for 5 seconds and plantarflexed for 5 seconds, one leg at a time.

0 = No visible tremor

0.5 = Questionable or barely visible tremor that could be normal

1 = Postural rhythmic tremor greater than 1 cm in amplitude (assessed at the foot). (many people “bounce” so need to emphasize rhythmicity

26) Trunk tremor while patient is seated on a table or stool with no support of the back.

0 = No visible tremor

0.5 = Uncertain (e.g., with severe head or limb tremor transmitted to the torso)

1 = Definite abnormal tremor that appears to emerge from rhythmic muscular contractions in the torso

27) Bradykinesia manifested by slow hypophonic speech, paucity of spontaneous movement, micrographia, facial masking or other slow movements that are reduced in amplitude (e.g., when walking).

0 = No bradykinesia

0.5 = Uncertain, could be normal variant

1 = Definite bradykinesia

Syndrome diagnosis (IPMDS-classification): _______________________________________________

Rules for scoring items 21 and 24:

STEA item 21 was scored 0 when the patient had no rhythm interruptions in three trials of rapid alternating movements with each hand; a score of 0.5 was assigned when the mean interruptions in 3 trials for either hand was >0 and <1; and a score of 1 was assigned for ≥1 mean interruptions for either hand.

Item 24 was scored 0 for no missteps, scored 0.5 for a minimum of 1-2 missteps in at least one of the 3 trials, and scored 1 for a minimum of >2 missteps in at least one of the 3 trials.

1. Regularity of head or hand tremor covers a large spectrum. Fully sinusoidal tremor is rare; position-dependence and/or jerkiness are very common. Jerky tremor is characterized by arrhythmic or irregular amplitude variations. [↑](#footnote-ref-1)
2. Low-amplitude jerks (mini-jerks) of the head or hand are sometimes seen isolated and should then be rated under item 5 or 10. [↑](#footnote-ref-2)
3. Unusual posturing, suggestive of dystonia, can be observed in patients with tremors. Some researchers consider this as dystonia and declare this to be proof of dystonic tremor. Therefore we need descriptive terms for phenomenology. [↑](#footnote-ref-3)
4. Missteps: Defined as a step during tandem gait which is more than half of the foot-width outside the gait line (left side: normal; right side: abnormal).

 [↑](#footnote-ref-4)
