# Supplementary material for: Exploring Interrater Disagreement on Essential Tremor Using a Standardized Tremor Elements Assessment
Source: Mov Disord Clin Pract. 2021 Feb 12;8(3):371–6. doi: 10.1002/mdc3.13150 (PMC8015892; doi:10.1002/mdc3.13150)

**MULTICENTER VIDEOTAPE EXAM PROTOCOL FOR TREMOR (draft June 25, 2018)**

**General Instructions**

 The purpose of the video is to document diagnostic features and severity of tremor. Also, additional symptoms (e.g., dystonia, myoclonus, ataxia, peripheral neuropathy) should be convincingly demonstrated.

 Make sure there is enough light in the room.

 If possible, have two people perform the recording. Keep the camera close to objects to increase resolution.

 Position camera to the region of interest as closely as necessary. Try to avoid zooming whenever possible because this worsens resolution.

 Remove glasses, if needed. Remove any food items or chewing gum from mouth. If special aspects need to be shown (e.g. toe tremor), make sure that the tremor can be seen appropriately.

 Video should be taken from straight in front of the participant, except where stated otherwise.

 Avoid stating or including in the video the participant’s name, date of birth, or other identifiers.

 Use a chair with comfortable arm-rest

 Record entire video in one clip if possible or on short sequences that can be put together. Use a tripod. Avoid dragging the tripod on the floor.

 Record the following statement at start of each video: “**This is subject Dys#X enrolled at (site name) on (current date & year).**”

**Part I: Head and neck tremor demonstration. Participant is seated in a chair without head support. Feet are resting flat on floor and hands are resting on the armrest. Film the entire body for 10 seconds. Focus camera to capture head and shoulders only (preferably by moving the camera and not by zooming). Make sure to reduce background noise, and use a microphone if possible.**

1. Document head tremor while the patient looks directly at the camera. Eyes open and closed for 10 seconds each. At the same time document facial tremor or hyperkinesia. Turn head comfortably (about 45 deg) left and right for 5 seconds each.

2. If head tremor or dystonia is present, demonstrate effect of a geste that is reported by the patient. (repeat in supine position, see below)

3. If present, document tongue tremor. Stick tongue out as far as possible and hold for 5

seconds; tongue at rest for 5 sec. Make sure there is enough light in the mouth

4. If present, document palatal tremor. Show the edge of the palate with the wall of throat and roof of palate (usually different angles are needed). Make sure there is enough light in the mouth

5. Let the participant read or read yourself and ask the participant to repeat aloud:

We mow our lawn all year

We eat eggs every day

He had half a head of hair

The puppy bit the tape

Ameisen eilen meinen Weg entlang Anita aß am Abend einen Apfel Mein Müller mahle mein Mehl

Der Sommer kommt mit Sonne

6. Ask the participant to hold long vowel sounds “aaaaaaaa” and then ‘eeeee’ (German ‘iiiiiiiii’)

for 5 sec each.

7. If any other manifestations of tremor of the head or neck/throat region are present, document appropriately.

8. If there are signs of dystonia or the neck/head region, document by showing the most comfortable head position and the subjective straight head position (subjective vertical).

**Part II. Arm and hand tremor demonstration. Participant sitting on a chair. Front view to camera. Obtain high resolution by moving the camera rather than by zooming.**

1. Document rest tremor**.** View from straight in front. Arms relaxed on armrests or on the patient’s lap. Hands dangling loosely (e.g., over the edge of the armrest) with upper limbs relaxed. Forearms are pronated in a relaxed posture. The forearms should not be actively supinated because the forearm muscles are then not at rest. Ask participants to completely relax the upper limbs and to count backwards from 100 or to state the months of the year backwards, starting with January. If rest tremor develops, ask participant to extend hand and fingers horizontally to document rest tremor suppression if present (if necessary second trial with higher resolution).

2. Document action tremors. View from straight in front.

a. Postural tremor. Extend arms/hands supinated towards camera (5 sec). b. Postural tremor. Extend arms/hands pronated towards camera (5 sec).

c. Move the hands up and down for 5 sec at a slow speed in an extended arm position.

d. Wing-beating tremor: Flex elbows and hold hands/arms horizontally in front of chest for 10 seconds. Fingers should be extended and spread apart. The hands should not touch any part of the body

e. Finger-Nose-Finger test to document intention tremor. Precision and accuracy should be emphasized. Avoid allowing the subject to arbitrarily move the limb from one target to another. The patient should be instructed to touch the examiner’s fingertip and tip of the nose as precisely as possible.

f. Document trunk tremor while patient is seated with back unsupported and while standing. g. Document postural tremor separately for each leg for 5 sec by extending the leg/knee

while the patient is seated. Legs are lifted separately to a horizontal position, and the toes

should be pointed upward for a few seconds and then distally for a few seconds, toward the hand of the examiner.

3. Document bradykinesia, rigidity, ataxia and entrainment

a. Finger tapping 5 sec each hand (thumb against index finger as big and as fast as possible)

b. Rapid alternating hand turns for 5 sec: upper limbs should be extended horizontally in

front of patient, and patient should be asked to fully pronate and supinate the hands as rapidly as possible

c. Rapid alternating pronation/supination of one hand into the palm of the other at a comfortable elbow angle of 90°, 10 turns in approximately 7 seconds. Repeat trial if first trial is abnormal.

d. Demonstrate rigidity and possible co-contraction (test each hand separately for hand rigidity)

e. *If functional tremor is suspected:* Perform an entrainment maneuver. Ask patient to hold the trembling limb in a position that produces tremor. With the opposite limb, the patient is asked to perform repetitive movements in unison with those of the examiner, who will vary the frequency above and below the frequency of the tremor. The examiner must correct the patient when the desired rhythm is not achieved.

f. Repetitive leg movements. Repetitively raise and lower the heel (not the ball of the foot) to

the floor approximately 6 cm, and do this 10 times in 5 seconds.

**Part III. Patient seated at a desk that can be properly filmed from the front or with a 45° angle of the participant, both hands above desk (participant may put glasses on at this point):**

1. Write cursively “This is a sample of my best handwriting” (“Das ist ein Beispiel meiner besten Handschrift”) with dominant hand on an empty sheet of paper. Secure the paper on the table if necessary. The patient should not try to steady one limb with the other.

2. Draw the large and small spirals of the Fahn-Tolosa-Marin scale with right hand and then with the left hand. Only the ballpoint pen should touch the paper, not the hand or forearm. Use the Fahn-Tolosa-Marin spiral templates. The spirals should be drawn starting at the center. Patient should make no attempt to brace the arm against the torso.

3. Display the sheets with writing and spirals briefly in the video.

4. Document any dystonic postures that occur during these tasks.

**Part IV: Participant stands. Zoom camera out further to capture entire body, including head and all limbs**

1. Document balance difficulties. Standing with both feet ~30cm apart for 15 sec, standing with both feet together, eyes open for 15 sec and 15 sec with eyes closed.

2. *If tremor during standing is present*: Show tremor of the legs or hands during standing.

Demonstrate tremor features when present during standing on the toes.

3. *If orthostatic tremor is present*: Document stance time. Patient standing before a chair (to get seated if endangered) with the feet together and to stand as long as possible. Measure and document stance time until participant needs to move or to sit down.

4. Ask the patient to walk 10 m, turn around, and walk back toward the camera (2 times).

5. Ask the patient to walk 10 tandem steps. For each step, the heel of one foot must touch the toe of the other. Repeat this task up to 2 times if the first trial is abnormal.

**Part V: Demonstrating extremity, head and voice tremor in a supine position. Tremors are evaluated in a supine position with the participant relaxed on an examination table, with full support of the head and extremities. Camera on the region of interest. Roll pant legs up to the knees. Remove pants if necessary. Patient should be barefooted.**

1. Document upper and lower limb tremor at rest while supine. Ask the patient to count backwards or state the months of the year backwards, beginning with January. If rest tremor is present document suppression during movement of the limb.

9. Document kinetic lower limb tremor: Heel- knee-shin test with the right and left lower limbs.

The task should be performed barefooted. The patient is instructed to move the heel lightly and precisely on top of the shin, between the knee and ankle, 3 times. The heel-knee-shin task should be performed without interference by clothing.

2. Document head tremor at rest and during posture. Ask to lift the head from the pillow. Ask the subject to bring the head in the least trembling position.

3. *If voice tremor present repeat at rest*: “aaaaaa” and “eeeeeee” for 5 sec.

**Part VI (if applicable): If participant has a task specific tremor or dystonia not captured in the preceding tasks (such as playing musical instrument, chewing, etc.), please videotape the dystonic symptoms while participant performs such tasks! Show all tremor manifestations that are not mentioned here (focal tremors, orthostatic tremor), including the task that most**

**strongly expresses the symptom and the task that most strongly suppresses tremor. In case of dystonia, postural abnormalities should be shown during standing, and facial dyskinesias during action (eyes opening and closing, smiling, etc).**

**Part VII (if applicable) Document signs of neuropathy (Atrophic skin changes, paresis, high- arched feet, hammer toes, muscle atrophy). Document other general neurological abnormalities if present.**

Dominant Hand


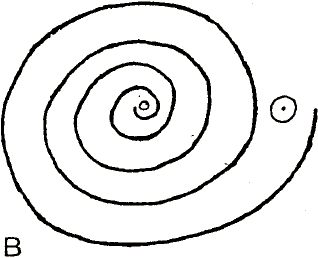

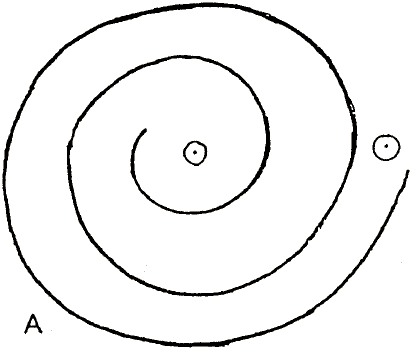


R *I* L (circle one) Nondominant Hand


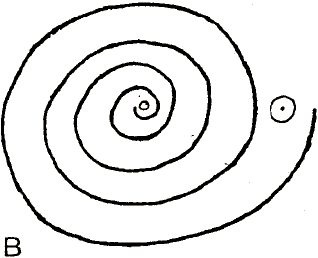


R *I* L (circle one)


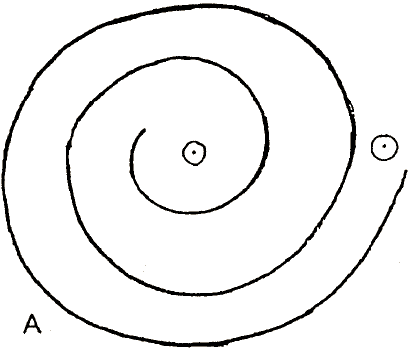

Supplement: Supplementary file 3 — Supplementary 2. Videotape exam protocol for STEA. [file MDC3-8-371-s002.docx]
